# A Sanitary Crusade against Cave Dwellers

**Published:** 1919-06

**Authors:** Manton M. Carrick


### A SANITARY CRUSADE AGAINST CAVE DWELLERS.

By MANTON M. CARRICK, M. C.

* An address to the soldiers delivered at Camp Holabird, Baltimore, Maryland, February 19th, 1919.

There is an old-time fable of a man who having dwelt many years in a dark cave, grew weary of it, and began to pray for the sun to come and remove the darkness. "Darkness?" said the sun, "What is darkness?" The man replied, "If you will only come and look into my cave you may see for yourself." So the sun came and looked into the cave, its rays penetrating to the darkest recesses. "Darkness? Where is the darkness?" was the demand.

There is a parallel in this enigma for you men. May I not help you to find it? Do you not realize that countless men and women are still living in caves,—cobwebby caves of superstition, ignorance, uncleanliness, disease and despair, wandering aimlessly through life, broken cogs in the great wheel Do you not realize that they are praying for the sun to come and banish their darkness?

It is you men who are the great orb that have it in your power to flash the light of your knowledge and understanding into their caves of ignorance and cheerily say, "Darkness? What is darkness?" And by so doing thousands of deaths, thousands of cases of serious illness will be obviated, while thousands of instances of increased health, tone and efficiency will result.

Here is the situation in a nutshell. The war is over. You men will soon be discharged and scattered. The majority of you will shortly return home to become readjusted to civilian life once more. For months now you have been living under military discipline. Uncle Sam has been doing your thinking for you from the first moment, when the Power which upheld the maxim "Might makes Right" disregarded the will of the people and plunged us into a world war. "It is all over now," we say, but the thought that I wish to bring home to you men is, that though the armistice is signed your responsibilities are nowise lessened, but increased if anything. The War of Ages is at our doors for you to fight with all your red-blooded young manhood,—cave dwellers are crying to be liberated from their darkness; there are scourges of disease caused by insanitation, the great White Plague, typhoid and para-typhoid fevers, small pox, hookworm disease, chronic malaria and worst of all, the Social Evil. What are you going to do about it? It is up to you, in the vernacular of the man on the street, to help rid our fair land of these blots on her escutcheon by demanding their eradication. Will you do it?

"They shall not pass!" cried that brave band of soldiers who held the field against their treacherous foe. Why not take this classic phrase, destined to go down the centuries into history, for your slogan in your warfare against the enemy battering against the doors of civilization, your own doors, my soldier friends?

How can it be done? By each returning soldier doing his "bit" to help mould public opinion in his own civilian community, not only by his own lofty life of citizenship, but in such a masterly manner as to create a demand for the enactment of laws relative to the matters of health and their enforcement when they are once placed upon the statute book.

What was it that made your camps fit places to live in? Sanitation. You all know that the war had no sooner been declared than Surgeon General Gorgas got busy. "It is the business of the Medical Staff to keep our men well," he said with a snap of his jaws that meant just what his lips uttered. Accordingly, safeguards, moral, mental and physical, were thrown around you men destined to figure in the greatest war the world has ever known. Never in the history of the universe have such sanitary precautions been taken. You know all about it. I do not need to tell you how your camps and the communities in which they were stationed were made as perfect as modern science could make them, in order to prevent the spread of communicable diseases and to keep you in health. Despite all the Herculean efforts put forth the great scourge of influenza played its havoc with soldiers as well as civilians. But the fault was not with sanitation. Unfortunately science cannot always work as rapidly as disease germs. Had it not been for these same sanitary measures, however, most of us would not be here today to tell the tale.

During your months of military training you men have acquired a knowledge of the fundamental principles of sanitation which should be valuable to you and the communities to which you are soon to go. You all know the importance of cleanliness of body and surroundings; the danger of overcrowding; of direct contact with communicable diseases; of insufficient ventilation and the unnecessary exposure during periods of temporary impaired vitality. You know, too, the proper corrective measures. Then, will you do Uncle Sam the honor of making use of this knowledge? Of shining into the caves of darkness and dispelling the gloom?

When you go home it is going to be hard at first to readjust yourselves to the old life. It will not be so noticeable in the first joy of being back, but after a time you are going to be restless. You have seen something of the world and have a different viewpoint than the folks back home. As a matter of fact "Private Jim" is quite a different person from the old Jim Smith. The home folks are just the same, of course, and you are going to love them exactly the same, but—well—it will be different. To them you will be "Bill" and "Jim" and "Jack" instead of Sergeant and Corporal and Private, but you will also be something else,—a hero, a little bit better than the common clay in those parts, set upon a pedestal to be worshipped. Now all this will bore you a bit to be sure, but you have got to live up to it. I tell this, Sergeant Bill, Corporal Jim and Private Jack, because I want you to realize that in justice to Uncle Sam and yourselves you MUST live up to it, for that is what is going to save the day for you, your Uncle Sam, and the home-folks who are just aching for your return.

It is you who are different, not they. You are the one who will have to do the most readjusting, but do you know what is going to be your salvation and prove you to be the hero they believe you to be? To love your old home town well enough to take an active, vital part in everything that concerns it. Perhaps you are naturally modest or diffident about thrusting yourself forward. You will more than likely say to yourself, "I don't seem to want to know it all because I've been a soldier. . . They'll get on as well without me." That won't be the right attitude. Your town does need you, the best you have to give, and you need it, if for nothing else than a safety valve in your own individual readjustment.

You were not a "Slacker" when it came to enlisting. You are not going to be a "Slacker" now.

Do you realize that a large proportion of the diseases from which mankind is suffering are preventable? That they are due to social rather than individual offenses, and that the remedy must be found in the social organism rather than the individual? That the improvement of hygienic conditions in your city, town or rural community requires a certain amount of specific regulation, and that while health officials may point the way and lead the fight they cannot accomplish much without the public? You, in this case, are an atom of the public and can help in its education perhaps more than you realize.

First of all, begin in your own home to mould public opinion. Your mother may be the best housekeeper in the world, and to all appearances everything be spic-span, and yet unsanitary. You are in a position to know if this is so, and to tactfully remedy the defects. As a matter of fact you will be in a position to do and say what no stranger would dare attempt. When the family sees that you take warm daily baths, followed by a cold splash, plunge or shower, according to facilities, that you are more particular about your clothing and its care than before you went away, that you are particular about the ventilation of the house and your own room, that you avoid badly ventilated, badly lighted, dirty, overcrowded and damp rooms, they, too, are going to ponder over these things.

You know the danger arising from breathing dust; that it is a disease-breeder. Then why not discourage the old tacked-down carpets if they are still in use in your home, oil the floors and make your mother a present of an oil mop? Then if you are in doubt about the drinking water, why not boil it? Some corner pumps are dangerous, you know, and even old oaken buckets while picturesque and interesting to carol about, are apt to be reeking with germs—myriads of them. Perhaps you are suspicions of your dairyman and the milk he is leaving at your door. If so, quietly investigate him and learn if he has a clean, sanitary dairy.

No one will even suspect you as a crusader at first, if you are "Wise as a serpent and harmless as a dove." You can just be good naturedly helpful in and out the house spare moments, cleaning up and planting the back yard screening the house especially the kitchen and by all means the porch, in order to keep out that disease and death-breeder, the filthy fly, as well as his partner in crime, the mosquito. Meantime, you can appoint yourself custodian of the garbage pail, burning all waste matter in which flies or mosquitoes might breed.

In a short time you will notice that the entire neighborhood is following your example. A clean-up campaign even on the most modest scale is always as contagious as whooping cough or measles.

Perhaps your home happens to be in a community which prides itself on its irrigation facilities, the sort that makes the desert blossom like a rose and at the same time produces a crop of malarial-breeding mosquitoes. If so, you indeed have a problem to conjure with, but I am sure you will be quite capable of solving it. You can educate your community to a realization that mosquitoes are more than a troublesome pest, that they are responsible for many deaths, much human misery and a great economic loss through their activity as disseminators of malarial and yellow fevers. You can call to the attention of individuals, and also the public through letters addressed to your daily papers, the pernicious activities of insects in their relation to disease, appealing to individual effort and community cooperation to get rid of this pest. Point out that professional zoologist, medical men and laymen alike know how detrimental insects are to public health, and that insect-borne diseases are a constant menace to the community. Make it quite clear that with the exception of the rat-flea, no insect can compete with the mosquito in its deadly work.

Drop hints as to modern methods of ridding a community of this pest and its breeding pools, by drainage, changing the water course, filling in and oiling streams; of the importance of screening in order to prevent infection by mosquitoes; medical prophylaxis; of immunization of a susceptible popluation. Make it quite clear from the beginning that malaria responds quickly to anti-mosquito work and quininization, once the mosquito-breeding places are eradicated.

Do not hesitate to disseminate all the knowledge you possess about the housefly, his origin in man's feces, or horse-manure, Milady Fly's chosen places for bringing forth her young, though she also revels in rotting vegetables and filthy corners of any sort. Tell the story of the chain of events through which the housefly may infect food with bacteria or other organisms derived from excreta; how flies are an important factor in disseminating typhoid fever through both food and milk, et cetera. This is your opportunity to open the eyes of the public to the grave danger of open privies in the vicinity of wells and vegetable gardens, thus contaminating the soil; of manure bins open to flies; the danger of garbage being placed in open containers instead of being burned or placed in covered sanitary pails. Here, too, is a chance to say something about the prevention of waste and the utilization of by-products like garbage for soap-fats.

Since the war began there has been such a shortage of fats that shrewd men have gone into the business of making soap fats from garbage. In fact, there will be such a shortage for several years, so an opportunity is offered the thrifty along new lines. These new garbage reduction works now operated in the country save about $8,500,000 worth of grease and $2,500,000 worth of fertilizer. They also produce about 9,000,000 pounds of nitrogen, 25,000,000 pounds of bone phosphate-lime, and 2,500,000 pounds of potash. It is an endless chain, but this is enough to show you something of waste prevention. The rest you can easily prove for yourself if interested in writing the Department of Agriculture for some bulletins along this line.

In short, you can gently lead the entire community into paths of sanitary righteousness without their having any idea of what you are doing. You can refuse to patronize, or take any of your friends to an ice-cream parlor, or soda-water fountain that you know to be a typhoid-fever dispensary as so many are, owing to the lack of screening facilities, and conveniences for washing glasses and dishes.

Last but by no means least, you have seen the great advantages of innoculation against typhoid and para-typhoid fevers; of vaccination for small-pox; of the recognized treatment for the elimination of hookworm; of the great desirability for proper medical attention in cases of chronic malaria. These are irrefutable facts in our army and you are now privileged to return to your various civilian communities, many of which are prejudiced against vaccination through lack of knowledge, ignorance or superstition, and to lift the veil of darkness in their caves. You can emphasize the desirability of these modern preventative measures, and urge that they be employed. By the same methods that you have made your crusade along sanitary lines, you can direct public attention to these irrefutable facts, and thus save entire communities from terrible scourges.

Can you not see, men, how our hope for the future lies wholly in you? It is now up to you to make your homes better places to live in; to lower the death rate in your individual communities and to make them more wholesome in every way. With such service rendered your country there will come a fine sense of well-being, a better moral tone and a keener realization of the joy and art of living. Let our light shine!

